# On Clinical Utility and Systematic Reporting in Case Studies of Healthcare Process Mining. Comment on: 10.3390/ijerph17041348 “Towards the Use of Standardised Terms in Clinical Case Studies for Process Mining in Healthcare”

**DOI:** 10.3390/ijerph17228298

**Published:** 2020-11-10

**Authors:** Dylan A. Mordaunt

**Affiliations:** 1 Shoalhaven Hospital Group, Illawarra-Shoalhaven Local Health District, Nowra 2541, Australia; d.a.mordaunt@gmail.com; 2 Faculty of Medical and Health Sciences, University of Adelaide, Adelaide 5005, Australia; 3 College of Medicine and Public Health, Flinders University, Adelaide 5042, Australia; 4 School of Medicine, University of Wollongong, Wollongong 2522, Australia

Recently in *Environmental Research and Public Health*, Helm and colleagues reported on a systematic review of healthcare process mining (HPM) case reports, focusing on the reporting of technical and clinical aspects and discussing standardisation terms in future HCM reports utilising existing ontologies [1]. HCM remains in its relative infancy, necessitating a shared understanding of terms and concepts as the wider community begins to leverage these techniques.

In healthcare, there are numerous processes often framed differently—patient journey, clinician and administrative workflows, prospectively designed organisational/clinical pathways, supply chains, etc. Since these processes are highly dependent on individual patients, clinicians, and administrators, as well as the interface between multiple other processes and systems, there is considerable variability in many pathways. “Warranted” clinical variability occurs where a deviation in the usual pathway occurs because the patient/condition warrants differential management. In contrast, “unwarranted” clinical variability is seen as a critical factor associated with suboptimal health outcomes [2,3,4].

Helm and colleagues highlight the importance of the semantics of healthcare jargon, that is, terms that might be widely used but have different meanings in specific contexts. Indeed, this problem is widespread within healthcare, where terms can have different meanings for different individuals, particularly acronyms—a simple example being ASD, which can mean *Autism Spectrum Disorder* in one context, or *Atrial Septal Defect*, *Acute Stress Disorder*, *Anti-Seizure Drug*, *Arthroscopic Subacromial Decompression,* or *Aspartate-Semialdehyde Dehydrogenase* in another. Further exacerbating this problem is that not all terms in everyday use, in every language, are reflected in terminologies and meta-ontologies; some terms are reflected in multiple ontologies that have different use-cases.

Even “problems” or “diagnoses” may have different implications for how they are used. A patient with developmental difficulties may be placed on an Autism Spectrum Disorder pathway, found to have speech delay but not to have Autism Spectrum Disorder. They may continue on that pathway as a function of the healthcare system, and indeed in some systems they may even attract a diagnosis of autism spectrum disorder in order to access funding or interventions such as therapy [5]. This context is essential for us to understand the generalisability of the findings of a study to a particular setting, some of which might be taken from parallel knowledge of an environment, however much of which would ideally be either included or referenced within a report, to maximise an understanding or translatability and generalisability.

Furthermore, in secondary re-use of clinical and administrative data, it is essential to address the purpose for which the data was primarily obtained, who produces different elements of the data, and what the data quality issues are that might result. In Australasia, the International Classification of Diseases (ICD) codes are primarily used for funding and planning purposes and not for primarily clinical purposes. Inpatient care, surgery, and first episode of cancer tend to coded by a group of professional clinical coders; outpatient episodes do not tend to be coded at all, since funding is time- and service-based. In contrast, the United States has widespread clinician coding. In both environments, policy changes intended to improve healthcare quality, outcomes, and ‘’value’’ have had a significant impact on coding, for instance, in the form of coding intensification (subsequently mitigated by various risk adjustment methods) [6,7].

ICD code usage is particularly problematic outside of funding and planning, and is dependent on the unique code and the particular environment. In the South Australian experience of re-designing state-wide stroke pathways, it was found that ~25% of codes were incorrect. This may be suitable for most current process mining work. However, it is essential to recognise the quality issues that may arise in a particular environment and both pre-analytical and analytical attempts made to address them [8]. If we were to assess the pathway of patients with achondroplasia undergoing ventriculoperitoneal shunt insertion, under ICD-10 codes they would be lumped into the same category as hypochondroplasia, which is a different condition in which hydrocephalus is far less frequent a problem. ICD code usage in some health informatics applications is so problematic as to render the labels practically unusable. For example, in analysing the ChestXray14 dataset [9], Oakden-Rayner identified that the correlation between ICD-10 labels and the radiology image findings was low [10].

Helm and colleagues point to the lack of a universal ontology for describing healthcare speciality. Indeed, even within a single “speciality”, healthcare is planned and delivered by several practitioners that influence the outcome. From a regulatory basis alone, what constitutes one speciality or discipline in one country often is not the same definition as another country. This domain is so heterogeneous that rather than a hierarchical ontological approach, it may be more helpful to have multiple descriptors of the domain—the types of practitioners involved, their speciality, the range of experience, a description of the setting (country, city, and dominant ethnicities), etc. Some of these may have corresponding SNOMED CT or meta-ontology terms; however, a structured analysis could limit this to terms that map with a corresponding subset. For instance, in New Zealand and the United Kingdom, healthcare speciality is defined by the Medical Council of New Zealand (MCNZ) and General Medical Council (GMC), respectively, rather than by SNOMED CT. Thus mappings could be made to this ground truth (NZ defines 38 specialities).

Helm and colleagues have highlighted the need to converge on a framework for describing features of an HCM case study, and have identified areas for reporting. Reporting guidelines are well established in several areas of healthcare, particularly those by Enhancing the QUAlity and Transparency Of health Research (EQUATOR) network [11]. Healthcare process mining is mostly observational and performed in retrospect, with secondary re-use of data collected for clinical and administrative (largely billing) purposes rather than for primary business process management (although this is not always the case, e.g., [12]). Some useful frameworks that share similarities might be the Transparent Reporting of a multivariable prediction model for Individual Prognosis Or Diagnosis (TRIPOD) Statement [13], The Strengthening the Reporting of Observational Studies in Epidemiology (STROBE) Statement [14], and ongoing work related to producing similar guidelines for reporting machine learning models [15].

Clinically, we generally start with a question or a problem description. Direct and indirect methods of describing population health problems and questions are well described, and commonly use the Population, Intervention/Exposure, Comparison, and Timing (PICO(T)) framework for framing a question. Indeed, attempts to automate information extraction from biomedical literature have leveraged this framing [16]. So, in addition to the elements about adopting the use of standard clinical descriptors, some essential caveats warrant expanding on the terms/codes included in core ontologies where possible. In terms of encounter environment or context, the PICO framing expands this to: what was the source population, and who were the participants that were included? Were there any essential individuals or cohorts explicitly or implicitly excluded? What was the source system, and what was its primary and typical secondary uses? Are there known problems with the data? Is there anything specific to the environment that is unique? Were there particular observations taken or interventions administered during the process? What are the essential outcomes of the process, either process, proxy, or not?

Many insights remain to be learned from HCM in clinical usage. Many of the published case studies focus on technical aspects of discovery, which is understandable given we are still coming to grips with understanding the technology in healthcare and since the tool developers/users are potentially the ones seeing many of the opportunities. For us to leverage this clinically, we need to understand how the insights are useful, how to leverage them to plan and influence changes to processes, and how the insights can be translated into practice. For example, Kempa-Liehr et al. [17] recently published work on surgical pathways, including cholecystectomy and appendicitis in which they identified several branch pathways, i.e., clinical variability. The extent to which these pathways were “real” or not remains unclear, and so even rare branches may be informative for rare events. In contrast, common branches may relate to nosological or data quality issues. Determining which was warranted and unwarranted required more than clinical input since the clinical executive and governance often decide on what the endorsed patient pathway is supposed to be.

So, how we use the mined data in clinical and administrative practice, in terms of translation, remains unclear. Is it merely a matter of health implementation science, acting on the insights to realise the benefits of understanding process variation or delays, and the art of influencing/change management, or is a broader issue that we do not yet fully understand the full power/utility of the insights? Many case studies seem to have been performed on similar pathways. Systematic reviews have focused on various aspects, such as the domains studied, and in this recent report, aspects of data quality. As yet, comparative process mining seems to be limited, i.e., a comparison of these processes between environments, or more importantly, across hospital systems. Could we link the variations in pathway/process with health outcomes (except for process-related and proxy outcomes)? To understand this, we will need to bridge the knowledge gap for clinicians and health executives, in a similar way that is occurring in other technological domains such as artificial intelligence.

In summary, Helm and colleagues focused on systematising aspects of the HCM case study reporting. There are problems with existing terminologies/ontologies/coding systems and limitations to solely rely on these. There is a precedent based on similar guidelines for a structured guideline or tool to be developed that will guide reporting and encourage standardisation. In addition to the aspects Helm et al. reported, it would need to cover core clinical/epidemiological/administrative questions such as PICO, and reports would ideally discuss the clinical/business implications of the insights obtained from the modelling.
